# Supplementary material for: Association and Effectiveness of PAX1 Methylation and HPV Viral Load for the Detection of Cervical High-Grade Squamous Intraepithelial Lesion
Source: Pathogens. 2022 Dec 30;12(1):63. doi: 10.3390/pathogens12010063 (PMC9865608; doi:10.3390/pathogens12010063)
Supplement: Supplementary file 1 [file pathogens-12-00063-s001.zip › pathogens-2045363-supplementary.pdf]

Table S1. PAX1m and hrHPV viral loads relative to the grade of cervical lesion [median(IQR)]

Biopsy	N	PAX1m	Log(all-hrHPV)	Log(HPV16/18)	Log(other-hrHPV)
≤CIN1	157	18.6 (11.5-20.2)	2.88 (0.00-5.05)	0.00 (0.00-0.00)	1.51 (0.00-4.35)
CIN2	118	19.2 (11.1-20.7)	4.57 (2.99-5.67)	0.00 (0.00-3.88)	3.58 (0.00-5.09)
CIN3	171	9.9 (7.6-19.1)	4.68 (3.60-5.50)	2.82 (0.00-5.01)	1.85 (0.00-4.44)
SCC	30	5.7 (4.0-6.9)	4.76 (4.17-5.43)	4.29 (0.00-5.28)	0.00 (0.00-4.12)

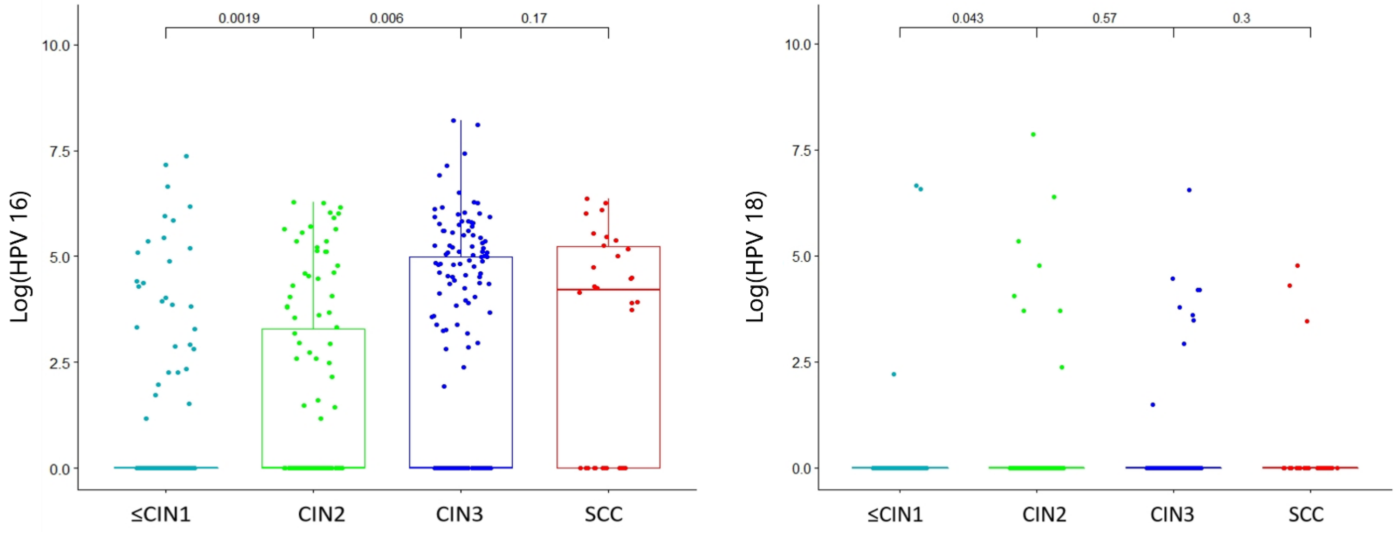

Figure S1. HPV16 VL (a) and HPV18 VL (b) relative to the degrees of cervical lesions.
